# Cultural context of professional identity formation: a perspective of health profession educators in Botswana

**DOI:** 10.3389/fmed.2026.1916041

**Published:** 2026-10-05

**Authors:** Molefhi Abotseng, Enoch Sepako, Keikantse Matlhagela, Matshidiso Matome, Gaonyadiwe Lubinda, Ludo Badlangana

**Affiliations:** 1 School of Biomedical and Pharmaceutical Sciences, Faculty of Medicine and Health Sciences, University of Botswana, Gaborone, Botswana; 2 School of Nursing, Faculty of Medicine and Health Sciences, University of Botswana, Gaborone, Botswana

**Keywords:** Botho/Ubuntu philosophy, decolonisation, health profession educator, *Mabogo-Dinku* model, professional identity formation

## Abstract

In Botswana, the phrase “*motho ke motho ka batho—“*I am because you are” is culturally a way of life and core to who we are as a people. As Batswana, our relationships and the evolution of our individual and collective identities are built on this key concept of *Botho/Ubuntu*. For us, *Botho* dictates that one does not merely “become” but “becomes with” in relation to identity formation, and by extension, professional identity formation (PIF). In this paper, the authors share their perspectives on professional identity formation (PIF) for health professions educators in the context of scarce African and non-Western epistemologies in the literature. The authors' guiding question and focus is: are the existing models/frameworks of PIF contextually relevant? If not, how can they be enriched and contextualized? This is one of the key tensions in the PIF discourse. The authors contend that contextualizing PIF efforts must integrate holistic cultural lenses and local ontologies into the current PIF models. We illustrate how leveraging indigenous knowledge frameworks encapsulated in the *Botho* belief system could contextualize existing PIF frameworks using a model we have termed, “*Mabogo Dinku*”. The phrase *Mabogo Dinku* is derived from a prominent Setswana proverb, “*mabogo dinku a a thebana*”, meaning, working together towards a common goal. The model is in a pictorial form and highlights the interconnectivity between the different frameworks. The model suggests that if any of the frameworks are misapplied the whole *Botho* belief system weakens and so too the concept of “becoming with”.

## Introduction

Professional identity formation (PIF) is a dynamic lifelong process in which a person's existing identity is shaped by education, experience, relationships and reflection (1). Health professions education (HPE) is an integral component of PIF. We recognise that HPE is all-encompassing in that it includes students, educators and teaching. However, in this paper, the focus is on PIF for health professions educators. Educators need to have a strong foundational reflection of their professional identity formation since it influences the learners’ PIF.

The authors’ lived experiences, indicate that current PIF models do not fully reflect the non-Western frameworks that could enrich, challenge, and contextualize prevailing models of PIF (2). Additionally, non-Western literature in PIF is scarce (3). In this paper, the authors share their perspectives on PIF for health professions educators in the context of scarce African and non-Western epistemologies in the literature. The authors’ guiding question and focus is, are the existing models/frameworks of PIF contextually relevant? If not, how can they be enriched and contextualized? This is one of the key tensions in the PIF discourse. The authors contend that contextualizing PIF efforts must integrate holistic cultural lenses and local ontologies into the current PIF models.

In an attempt to address the existing tension in the PIF discourse, the authors propose a model, called *Mabogo Dinku,* which has the potential to contextualize and decolonize PIF through our cultural indigenous knowledge systems’ lens (Figure 1). The phrase *Mabogo Dinku* is derived from a prominent Setswana proverb, “*mabogo dinku a a thebana”.* This means, working together towards a common goal and that we navigate life much better through mutual aid, cooperation, and community support. The concept of *mabogo dinku a a thebana* is rooted in the *Botho* philosophical belief system. Through the *Mabogo Dinku* model we show that identity is multifaceted, nurturing and emerges through shared humanity. At the core of the *Botho* philosophy is the concept of “becoming with”. *Botho* provides culturally grounded relational perspectives that remain insufficiently represented within the current PIF literature.

**Figure 1:**
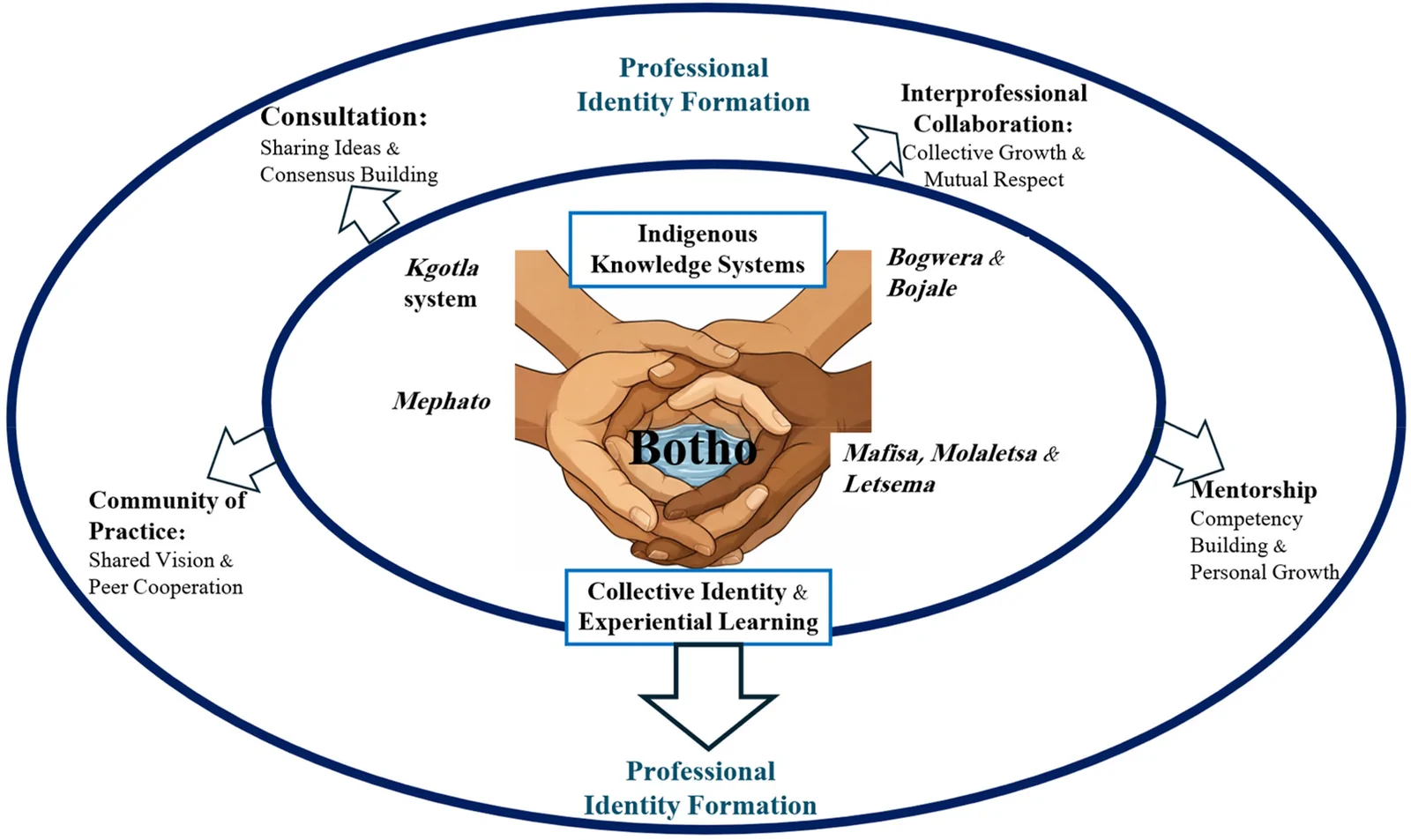
*Mabogo Dinku* – “Becoming with”– Model. In PIF, “*Mabogo dinku a a thebana”, is* working together to help each other to achieve a common goal. This Figure demonstrates how indigenous knowledge systems and cultural practices can feed into PIF. The inner circle elements are frameworks of the indigenous knowledge systems. The outflow from the inner circle is the collective identity and experiential learning which leads to the development of PIF. The image of the interlocking hands was generated with the help of artificial intelligence (AI).

We, the authors, write this paper as a multidisciplinary team of health professions educators from the University of Botswana (UB). UB was founded on the principle of “*Mabogo Dinku a a thebana*” (4). UB has its values focusing on “cultural authenticity by ensuring that diversity of Botswana indigenous values and cultural heritage forms an important part of the academic and organizational life of the institution” (5). However, the authors observation and lived experience is that there are limitations in translating these values as it relates to PIF in health professions education.

## Discussion

Professional identity formation is a complex and transformational process. It embodies the individual self-concept that is formed by a conversation between self and the outside reality in a social context (6). This is a concept espoused by social identity theory. This theory states how people define themselves in terms of group membership, for example, “I am a nurse”; “I am a doctor”; “I am a teacher” (7). It posits that an individual's self-concept derives from their membership in social or collective groups (8). Individuals adopt the prototypical behaviours, ethics, and norms of the professional group (9). This is where individuals categorize themselves into occupational groups. The discourse within the profession emphasizes in-group norms. For example, “this is how we talk, conduct ourselves, and think as a certain group of professionals” (10). However, critical approaches to identity compel us to consider among others, the intersectionality of history, culture, race, socioeconomic status and gender to arrive at a more nuanced and contextual understanding of identity (11). The authors contend that contextualizing PIF efforts must integrate holistic cultural lenses and local ontologies into the current PIF models. Therefore, considering our indigenous knowledge systems, the authors’ perspective is that PIF, in the context of *Botho,* is more than “who I am” and “who I want to become”. It is also about “becoming with”, which is the ultimate goal.

Presently, health professional educators are showing growing interest in PIF, viewing it as foundational to grooming and developing competent and ethical graduates (12, 13). Increasingly, PIF is seen as a hybrid identity that merges specific expertise with a broader collaborative interprofessional and transprofessional mindset (14). The authors have attempted to explain how our indigenous knowledge frameworks, rooted in *Botho*, could enrich, contextualize and decolonize PIF in the paragraphs below.

### *Botho: “Motho ke motho ka batho*”—“I am because you are”

The *Botho* philosophical belief system is expressed, nurtured, or fostered through African indigenous knowledge, cultural practices, and metaphors that convey the idea that collective effort is strength. Its value lies in the upliftment and the collective well-being of the community. *Botho* is integral to the fabric of Botswana (15). Consequently, as Batswana our worldview emphasizes collective achievement, where success is understood as a shared, communal outcome rather than an individual accomplishment. Hence, professional identity is formed through shared responsibilities, mutual support and is relational (16). This belief system has shaped our life experiences and unconsciously influences the authors’ PIF. Therefore, in our context, *Botho* cannot be ignored. Among the indigenous knowledge systems, social and cultural practices that emphasize collective achievement and shared communal outcomes are *mafisa, letsema, molaletsa, kgotla system, bogwera, bojale and mephato* (17–19)*.* We contend that PIF is shaped by influences like the community where you were born and the one where you grewup. These early environments instil values, resilience, and a sense of belonging that form the foundation of who you become as a professional.

### *Mafisa: “*I can only become with others”

*Mafisa* espouses the belief that wealth can be grown together and, by extension, represents the idea that, “I can only become with others” and, “we can go far together” (17). The *mafisa* is a traditional Botswana custom that builds social capital. Traditionally, it is a cattle loaning system where wealthy owners lend cattle to individuals without livestock, fostering mutual economic benefit (milk, draft power for the borrower; herd management/care for the owner) (20). This was frequently done among extended family members and relatives but was not restrictive. Notwithstanding, the fact that the *mafisa* system may have elements of patron-client hierarchy, traditional *mafisa* system functions in a symbiotic and mutually beneficial way: “as you grow, I grow too” (17). On the other hand, *mafisa* emphasizes interdependence and collective well-being. So, as we develop our professional identities, we tend to sideline *Botho* which is highlighted in our institution's policies, values and mission (5). Integrating *mafisa* as a cultural practice into PIF will strengthen and improve mentorship and interprofessional collaboration.

### *Molaletsa and letsema*: voluntarily working together to complete tasks

In communal cultural practices such as “*molaletsa*” and “*letsema*”, people work together voluntarily to complete heavy tasks that would be difficult for a family to achieve on time (17, 21). These practices are often used during the ploughing season for farming (*letsema*) and for harvesting (*molaletsa*). They are also used for building houses and weddings where the community will cooperate in solidarity and collective effort (20). These practices have shaped our cultural identities in Botswana, consequently influencing our PIF as educators. They are rooted in relational interdependence, resilience, and ethical reciprocity. These cultural practices involve ongoing “sense making” where personal events, relationships, and societal norms mould one's sense of “who I am” and “who I want to become” (22) and ultimately “who I become with”. It further dismantles the lone “wolf” mentality. It also integrates the personal, and professional lives into a cohesive identity.

### *Kgotla system* – consensus building

The traditional governance institution called the *kgotla* system epitomizes *Botho* and a collective shared governance irrespective of socio-economic status (18, 23). At the heart of the *kgotla* system is the idea that the elders, young people and everyone in the community gather in a circle of open dialogue. It is worth noting that the *kgotla* system as practised in Botswana has evolved and recognises historical exclusions or hierarchical relations. Traditionally societal structures were patriarchal. However, there are instances in the precolonial period (1600-1885) where royal women participated in decision-making and/ or became leaders (24). Post 1885, there were times where women became paramount/regents chiefs to their tribes (24). Currently in Botswana, five (5) tribes are led by women (25). These women serve as representatives of their tribes in Botswana's *Ntlo ya Dikgosi.* This institution bridges the traditional leadership and modern parliamentary governance (25). The *kgotla* system strives to be inclusive.

The *kgotla* system is grounded in five (5) principles: (i) neutral ground; (ii) no time limit on discussions; (iii) focus on the now and the future; (iv) collective responsibility and (v) proverbs (26). The system's commitment to hearing all viewpoints is highlighted by the ethos, “*mafoko a kgotla a mantle otlhe*”. That is, all viewpoints are beautiful or welcome (18). Furthermore, “*mmualebe o bua la gagwe*” meaning, everyone is allowed to freely share their perspectives, even dissenting voices. The voices of those present weave consensus from diverse perspectives, which is a timeless embodiment of *Botho*. This practice encourages everybody to participate in the public discourse since their voices are listened to, respected, and valued, even those that challenge the *status quo* (27). In some instances, poetry and songs are used to navigate and critique the actions, decisions and leadership style of the Kgosi (paramount leader of the community). This compels the leaders to confront uncomfortable realities. This is illustrated by the poems of Kgosi Tshekedi Kgama and Kgosi Molefi Kgafela (Leboko la ga Kgosi Tshekedi Kgama; Leboko la ga Kgosi Molefi Kgafela—published by Issac Schapera, 1965) (23).

Thus, the *kgotla* system has principles that could leveraged to create circles of open dialogue and consensus building. Interestingly, the *kgotla* system has found its way into the global corporate world. For example, a European consulting company named “*Kgotla*” (28), based in the Netherlands, uses the traditional operational systems and processes of the *kgotla system* for modern work environments. The authors’ view is that the institution has not fully embraced the principles espoused by the *kgotla* system. This skews authentic PIF and brings in tension where cultural identity and freedom of speech frameworks rooted in the *kgotla* system are not fully supported. In terms of PIF, the principles of collective responsibility and moving together into the future espoused by the *kgotla* system bring a sense that “you cannot become without becoming with”. The *Mabogo Dinku* model could provide an opportunity for institutions to strengthen the governance structures through open dialogue and consensus building.

### *Mephato, bogwera* and *bojale:* community building and mentorship

Another example of indigenous knowledge systems that highlight collective responsibility in community building and mentorship are in the form of regiments (*mephato)* and rites of passage (*bogwera* and *bojale)* events (19). These practices transform individuals from children into responsible, community-oriented individuals by instilling discipline, unity and a sense of shared responsibility (29). It is believed that the collective responsibility of the community is to mould somebody into a responsible member of the society. Hence, the African proverb “it takes a village to raise a child” (30). Given that *bogwera* and *bojale* are part of Botswana's culture, we can utilize this framework to mentor or to be mentored hence, “becoming with”. Mentorship is essential in PIF. High-ranking universities across the globe have rigorous mentorship programs (31–33). The authors’ experience is that formal mentorship opportunities at in our institution have been limited Therefore, the proposed “*Mabogo Dinku*” model has the potential of integrating the cultural ontologies with mentorship frameworks into PIF within the institution. This can be used to support our institution's aspirations of becoming a high performance organisation (5).

### “*Mabogo Dinku*” model: becoming with

The indigenous knowledge systems and cultural practices as epitomized in the *Botho* belief system described above indicate that a contextually based outlook of PIF can enrich the exisiting PIF models. In this regard, we propose a model termed “*Mabogo Dinku*” that can be used to achieve this (Figure 1). The model shows how the culturally grounded relational perspectives of *letsema, molaletsa, mafisa, bogwera, bojale*, *mephato* and the *kgotla* system can enrich existing approaches to PIF. *Botho* is the core of our culture and of the “*Mabogo Dinku”* model. Out of *Botho* emerges the foundational frameworks*: mafisa, molaletsa and letsema; kgotla* system*; mephato*; *bogwera and bojale.* These indigenous *knowledge* systems and cultural practices foster a collective, interconnected culturally grounded approach to PIF as depicted by the interconnected hands in Figure 1. There is overlap and interconnectivity between these frameworks. Specifically, the *mafisa, molaletsa* and *letsema* have aspects of interdependence, collective well-being and reciprocity which translate to interprofessional collaboration. In PIF these frameworks depict shared goals and clear roles with mutual respect. *Bogwera*, *bojale* and *mephato* have aspects of camaraderie and collective responsibility, experiential learning, interprofessional, community of practice and mentorship. For *mephato*, this interprofessional collaboration comes when individuals of different expertise come together to achieve a task. *Kgotla* system offers an open forum and safe space for a collective responsibility and of moving together into the future. It provides consultation and consensus building. If any of the frameworks are misapplied or utilized inappropriately the model weakens.

### Operationalization of the *Mabogo Dinku* model in PIF

The *Mabogo Dinku* model could be implemented within PIF leveraging the elements of the indigenous knowledge systems as summarized in Table 1. The authors propose an example of a guidance relationship between a professor and a group of early researchers. Here, the professor is integral in the professional development of the individuals under their stewardship. Thus, the values and skills to be developed are clearly defined and mutually beneficial. As an illustration, *mafisa* could provide a basis for symbiotic collaboration because it draws on the strength of each of the parties. *Molaletsa* and *letsema* could inform interdependence and multidisciplinary collaboration, hence breaking down silos in educational projects and research. The *kgotla* principles could inform structured dialogue, consultation, and consensus-building sessions. Finally, *mephato, bogwera and bojale* could inform peer, cohort or near-peer mentoring structures. When all these elements come together, as depicted in the intertwining hands of the *Mabogo Dinku* model, the individuals may receive professional advancement. Since *Botho* espouses the values of community upliftment as the ultimate goal, this professional growth leads to institutional excellence and improved ranking. However, we acknowledge that any system has limitations and pitfalls. Therefore, any implementation needs to consider these constraints and how the existing PIF models can complement the *Botho* belief system.

**Table 1:** Summary of indigenous knowledge systems and their operationalization in PIF.

Indigenous knowledge system	Contextual meaning	Application in PIF (operationalization)
*Botho* (overarching belief system)	I am because you are	Professional identity is formed through shared responsibilities, mutual support and is relational.
*Mafisa*	I can only become with others	Symbiotic collaboration, interdependence and collective well-being: “as you grow, I grow too”.
*Molaletsa and Letsema*	Voluntarily working together to complete tasks	They are rooted in relational interdependence, resilience, and ethical reciprocity: “who I become with”.
*Kgotla system*	Consensus building and collective responsibility	These principles of consensus building and moving together into the future could inform interdependence and multidisciplinary collaboration.
*Mephato, Bogwera* and *Bojale*	Community building and mentorship	Highlight collective responsibility in community building and mentorship are in the form of regiments (*mephato)* and rites of passage (*bogwera* and *bojale)* events.

## Conclusion

In this paper we attempted to answer a focal question on how *Botho* can enrich and contextualize current PIF models. In our context, *Botho* challenges us to reconsider our identity as inherently interconnected with the collective well-being of our community. The approach in PIF should be a contextual and relational accomplishment and an act of moving beyond ‘I’ to ‘we’, a collaborative pursuit of shared success and collective humanity. Therefore, integrating African and non-Western indigenous knowledge systems and cultural practices into PIF frameworks, can shape professional identity by grounding modern practices in local culture which, ensures that PIF is globally relevant but locally rooted. Professional constructs are not neutral in PIF, but are deeply shaped by holistic cultural influences, embedded values, power dynamics and worldviews from dominant epistemologies that define what is considered ethical, competent, and professional.

In conclusion, underrepresented epistemologies must be part of the PIF discourse, in order to enrich, challenge and contextualize prevailing models of PIF. Thus, a deliberate adjustment is paramount to merge global knowledge with local frameworks. This can be achieved by embracing and integrating indigenous knowledge into PIF. Through the *Mabogo Dinku* model, we have shown the possibility and potential of harnessing cultural practices to enrich and contextualise existing PIF models by using frameworks expressed in the *Botho* belief system. We acknowledge that we have not delved into the potential constraints of the indigenous knowledge systems. Therefore, in implementing culturally grounded frameworks to contextualize PIF models one must be cognisant of potential constraints.

In PIF, “*Mabogo dinku a a thebana”, is* working together to help each other to achieve a common goal. This Figure demonstrates how indigenous knowledge systems and cultural practices can feed into PIF. The inner circle elements are frameworks of the indigenous knowledge systems. The outflow from the inner circle is the collective identity and experiential learning which leads to the development of PIF.

The image of the interlocking hands was generated with the help of artificial intelligence (AI).

### Positionality statement

Becoming an educator is complex and involves interpretation and reinterpretation of who one is and who one wants to become in a multifaceted context. We are educators from Botswana, who grew up in rural Botswana. We attended our primary schools where cultural and traditional norms are still practised. At secondary schools, about half of the teachers were from around the world. Majority of us went to university in the western world. We then returned home and have been working in the University of Botswana ranging from 6 to 17 years of service. All these have influenced our view on PIF as we have experienced both the local and western influences**.** Therefore, we had to adjust and adapt to different contexts at a given time, and this has equipped us to be able to juggle and marry different paradigms. So, adaptation is ingrained in us.

It needs to be highlighted that UB has the responsibility of developing the highest HPE population. This shapes how staff and students interact and how PIF is shaped in both. Different institutional strata create distinct institutional contexts in which departments and units operate with their own norms, practices and cultures. Additionally, the university exists within a larger community which encompasses social networks, cultural norms, family influences, local practices and regulatory bodies. It should follow then, that the larger community should influence and shape the culture of the university. In this paper, we propose a model we have termed *Mabogo Dinku* to show how culture can be used to inform, enrich and contextualize PIF.

## Data Availability

The datasets presented in this article are not readily available because there are no data sets available to begin with. Requests to access the datasets should be directed to none available.

## References

[1] Wald HS. Professional identity (trans)formation in medical education: reflection, relationship, resilience. Acad Med. (2015) 90(6):701–6. 10.1097/ACM.0000000000000731 25881651

[2] Mokhachane M George A Wyatt T Kuper A Green-Thompson L. Rethinking professional identity formation amidst protests and social upheaval: a journey in Africa. Adv Health Sci Educ Theory Pract. (2023) 28(2):427–52. 10.1007/s10459-022-10164-0 36301374 PMC10169886

[3] Mende J Heller R Reichwein A. Transcending a western bias: towards a decolonised and entangled perspective in norms research. European Review of International Studies. (2022) 9(3):339–62. 10.1163/21967415-09030001

[4] Kwesiga E. Motho le Motho Kgomo. Dakar: Trust Africa (2024). Available online at: file:///C:/Users/abotsengm/Downloads/16.Motho-le-Motho-Kgomo.pdf (Accessed June 14, 2026)

[5] University of Botswana. University of Botswana Strategy. Gaborone, Botswana: University of Botswana (2025).

[6] Turner JC Hogg MA Oakes PJ Reicher SD Wetherell MS. Rediscovering the Social Group: A Self-Categorization Theory. Oxford: Basil Blackwell (1987). p. 239.

[7] Main P. Social Identity Theory: How Groups Shape Learner Behaviour: Cambridge: Structural Learning. (2026). Available online at: https://www.structural-learning.com/post/social-identity-theories (Accessed August 4, 2026).

[8] Stets JE Burke PJ. Identity theory and social identity theory. Soc Psychol Q. (2000) 63:224–37. 10.2307/2695870

[9] Cornett M Palermo C Ash S. Professional identity research in the health professions-a scoping review. Adv Health Sci Educ Theory Pract. (2023) 28(2):589–642. 10.1007/s10459-022-10171-1 36350489 PMC10169899

[10] Hogg MA Gaffney AM. Social identity dynamics in the face of overwhelming uncertainty. The Psychology of Insecurity. Routledge. (2023). p. 244–64. 10.4324/9781003317623-17

[11] Halualani RT. Doing” and “undoing” power as critical intercultural assessment: original teaching idea—semester for the core intercultural communication course. Communication Teacher. (2025) 39(2):117–26. 10.1080/17404622.2024.2414034

[12] Cruess SR Cruess RL Steinert Y. Supporting the development of a professional identity: general principles. Med Teach. (2019) 41(6):641–9. 10.1080/0142159X.2018.1536260 30739517

[13] Kellar J Austin Z. The Only Way Round is Through: Professional Identity in Pharmacy education and Practice. Los Angeles, CA: SAGE Publications Sage CA (2022). p. 238–40. 10.1177/17151635221115370 PMC9445508 36081919

[14] Frenk J Chen L Bhutta ZA Cohen J Crisp N Evans T. Health professionals for a new century: transforming education to strengthen health systems in an interdependent world. Lancet. (2010) 376(9756):1923–58. 10.1016/S0140-6736(10)61854-5 21112623

[15] Diplomat. “Botho”: the bedrock of Botswana’s being. Diplomatic Pouch. (2015). Available online at: https://diplomatmagazine.eu/2015/01/04/15776/20 (Accessed June 14, 2026).

[16] Orsmond P McMillan H Zvauya R. It's how we practice that matters: professional identity formation and legitimate peripheral participation in medical students: a qualitative study. BMC Med Educ. (2022) 22(1):91. 10.1186/s12909-022-03107-1 35139839 PMC8830078

[17] Resane KT. Mafisa: communion ecclesiology of acts 2: 42–47 in action. In die Skriflig/in Luce Verbi. (2025) 59(1):3234. 10.4102/IDS.v59i1.3234

[18] Tlou JS. Ubuntu as reflected in the kgotla system of the government in Botswana. Understanding Ubuntu for Enhancing Intercultural Communications. Hershey: IGI Global Scientific Publishing (2021). p. 1–8.

[19] Pheko BC Linchwe K. Leadership from two cultural perspectives—a tune or discord: Botswana's Experience. Int J Lifelong Educ. (2008) 27(4):399–411. 10.1080/02601370802051397

[20] Dube MW Modie-Moroka T Setume SD Ntloedibe S Kgalemang M Gabaitse RM. Letsema': Botho/Ubuntu: community building and gender constructions in Botswana. J ITC. (2016) 40(1):1–22.

[21] Lebeloane LD Quan-Baffour KP. ‘Letsema’: a way of inculcating and preserving African Indigenous Knowledge in the youth through formal education in the 21st century’. J. Educ. Stud. (2008) 7(2):43–9.

[22] Warhurst RP Black K Wu X-J. Reassessing reflection: theorising reflection as identity formation. Stud High Educ. (2025) 51(6):1–15. 10.1080/03075079.2025.2508825

[23] Lekorwe M. The kgotla and traditional leadership in Botswana. In Ray DI Quinlan T Sharma K Clarke TAO, editors. Reinventing African Chieftaincy in the Age of AIDS, Gender, Governance, and Development. Calgary, AB: University of Calgary Press (2011) p. 383.

[24] Makgala CJ Segobye A. A survey of women in Botswana history: individual pioneers, institutions and advocacy movements from the pre-colonial period to 2024. History Compass. (2026) 24(1):e70027. 10.1111/hic3.70027

[25] Botswana NyD. Members of the 5th Ntlo ya Dikgosi (2024−2029). (2025).

[26] Nkomazana F Tabalaka A. Proverbs, community building and governance: a critical analysis of the proverbial saying,“Kgosi ke Kgosi ka Batho” in Botswana. Scriptura: J Context Hermeneut S Afr. (2024) 123(1):1–12. 10.7833/123-1-2119

[27] Metz T. Duties to oneself in the light of African values: two theoretical approaches. Monist. (2025) 108(1):24–35. 10.1093/monist/onae027

[28] The Kgotla Company. Kgotla-Leadership Through Dialogue. Amsterdam: The Kgotla company (2003-2026). Available online at: https://kgotla.com/ (Accessed June 4, 2026).

[29] Matemba YH. Continuity and change in the development of moral education in Botswana. J Moral Educ. (2010) 39(3):329–43. 10.1080/03057240.2010.497613

[30] Reupert A Straussner SL Weimand B Maybery D. It takes a village to raise a child: understanding and expanding the concept of the “village”. Front Public Health. (2022) 10:756066. 10.3389/fpubh.2022.756066 35372232 PMC8964422

[31] Hochman J. Inspiring the next generation. Chicago: National Student Leadership Conference (2026). Available online at: https://www.harvard.edu/in-focus/mentorship/ (Accessed June 12, 2026).

[32] Burton PS. There are as many models of mentorship as there are mentors and mentees. Pretoria: Universities South Africa (2025). Available online at: https://usaf.ac.za/there-are-as-many-models-of-mentorship-as-there-are-mentors-and-mentees-says-professor-stephanie-burton/ (Accessed June 3, 2026).

[33] Noormahomed E Williams P Lescano AG Raj T Bukusi EA Schooley RT. The evolution of mentorship capacity development in low- and middle-income countries: case studies from Peru, Kenya, India, and Mozambique. Am J Trop Med Hyg. (2019) 100(1_Suppl):29–35. 10.4269/ajtmh.18-0560 30430979 PMC6329354

